# Insights into the Effect of the G245S Single Point Mutation on the Structure of p53 and the Binding of the Protein to DNA

**DOI:** 10.3390/molecules22081358

**Published:** 2017-08-16

**Authors:** Marco Gaetano Lepre, Sara Ibrahim Omar, Gianvito Grasso, Umberto Morbiducci, Marco Agostino Deriu, Jack A. Tuszynski

**Affiliations:** 1 Department of Mechanical and Aerospace Engineering, Politecnico di Torino, 10129 Torino, Italy; s214201@studenti.polito.it (M.G.L.); umberto.morbiducci@polito.it (U.M.); marco.deriu@polito.it (M.A.D.); 2 Department of Oncology, University of Alberta, Edmonton, AB T6G 2R3, Canada; siomar@ualberta.ca; 3 Istituto Dalle Molle di studi sull’Intelligenza Artificiale (IDSIA), Scuola universitaria professionale della Svizzera italiana (SUPSI), Università della Svizzera italiana (USI), Centro Galleria 2, CH-6928 Manno, Switzerland; gianvito.grasso@supsi.ch; 4 Department of Oncology, Department of Physics, University of Alberta, Edmonton, AB T6G 2R3, Canada

**Keywords:** p53, G245S-mp53, MD simulations, functional mode analysis

## Abstract

The transcription factor p53 is a potent tumor suppressor dubbed as the “guardian of the genome” because of its ability to orchestrate protective biological outputs in response to a variety of oncogenic stresses. Mutation and thus inactivation of p53 can be found in 50% of human tumors. The majority are missense mutations located in the DNA binding region. Among them, G245S is known to be a structural hotspot mutation. To understand the behaviors and differences between the wild-type and mutant, both a dimer of the wild type p53 (wt-p53) and its G245S mutant (G245S-mp53), complexed with DNA, were simulated using molecular dynamics for more than 1 μs. wt-p53 and G245S-mp53 apo monomers were simulated for 1 μs as well. Conformational analyses and binding energy evaluations performed underline important differences and therefore provide insights to understand the G245S-mp53 loss of function. Our results indicate that the G245S mutation destabilizes several structural regions in the protein that are crucial for DNA binding when found in its apo form and highlight differences in the mutant-DNA complex structure compared to the wt protein. These findings not only provide means that can be applied to other p53 mutants but also serve as structural basis for further studies aimed at the development of cancer therapies based on restoring the function of p53.

## 1. Introduction

The transcription factor p53 is a tumor suppressor protein that responds to cellular stress such as genotoxic damage, hypoxia, and other chemical or physical stresses, leading to cell cycle arrest, DNA repair and apoptosis [1,2]. In tumors, usually an abnormal cell cycle progression is observed due to activation of oncogenes or absence/defects in tumor suppressor proteins [3]. Indeed, *TP53* is mutated in more than 50% of human tumors. These mutations alter the function of the protein, which subsequently aids cancer progression and adversely affect patient survival. It is for this reason that many efforts have been made to find novel chemotherapeutics that target p53. Such examples include gene therapy, modulating the activity of p53 regulators and restoring the wild-type activity to mp53 [3,4,5].

Some 97% of p53 mutations occur in the DNA core domain of the protein [6]. One of the most frequent p53 mutants is G245S-mp53. This mutant is classified as a structural mutant. In other words, the mutation results in a conformational change that influences the binding of p53 to DNA and thus affects the protein’s transcriptional activity. The transcription factor, p53, binds as a tetramer to a double-stranded DNA consensus sequence containing two or more copies of the 10 bp half-site 5′-PuPuPuC(A/T)(T/A)GPyPyPy-3′ [7,8]. The mutation in G245S-mp53 is located in loop L3, which is involved in important interactions with the minor groove of DNA [8,9,10]. A zinc ion in the DNA binding domain (DBD) provides structural stability for the loops L2 and L3 and also has an impact on DNA-binding specificity [11]. 

It is difficult to pinpoint the exact effect of the G245S mutation on the process of malignant transformation and cancer progression. However, a study by Hanel and colleagues has demonstrated that while mouse embryonic fibroblasts carrying R248Q and G245S mutations were both transcriptionally inactive for p53 target genes such as *p21*, mice carrying humanized G245S-mp53, similar to p53-null mice, had slower tumor onset and death compared to mice with the R248Q mutation [12]. Also, the same study by Halen [12] and another by Xu et al. [13] have showed that Li-Fraumeni Syndrome patients with p53 G245 germline mutations had a later onset of cancer especially when compared to patients with p53 mutations in R248, which is also located in the loop L3 of the protein. However, in a different population-based study, G245 hot spot mutations were found to be correlated with poor prognosis and survival in colon cancer patients [14]. Understanding the consequences of mutations on the p53 protein structure may serve as the starting point for studies aimed at developing novel p53 targeted cancer therapies. Currently, APR-246 is the only p53 activator that is in clinical trials [4,15]; it can restore the transcriptional activity of p53 mutants, including G245S-mp53, to p53 target genes such as *PUMA* and *p21*. Stictic acid was also shown to restore the activity of G245S-mp53 to express *PUMA* [16]. In addition, it increased the thermal stability of the mutant protein in vitro [16].

Several studies which characterize the effects of hotspot mutations on the structure of p53 and its DNA binding properties have been reported. Previous studies based on X-ray crystallography and NMR spectroscopy have given an important insight into the biological structure of the wild-type p53 (wt-p53) (either complexed with DNA or free in solution, referred to as the apo form in this article) [8,9,17,18]. Experimental studies have shown that the wt-p53 core domain already has a relatively low thermodynamic stability (melting temperature of 42–44 °C) while G245S-mp53 has an even lower melting temperature of 39 °C suggesting a moderate destabilization of the structure [19]. The moderate destabilization of this structural mutant has been further confirmed by HSQC NMR spectroscopy where chemical shift changes between wt-p53 and the mutant were observed for residues in loop L2, L3 as well as terminal residues of the β-strands 4, 9 and 10, while the overall DBD structure folded as the wt-p53 [20]. As well, the crystal structure (apo protein) of the superstable mutant M133L-V203A-N239Y-G245S-N268D, a stabilized variant of the p53 core domain, suggests small conformational changes with respect to the wt in the immediate environment of the mutation site and in other key residues in the subunit interface of the core domain dimer bound to a DNA half-site [21]. However, none of the recent experimental studies have ever resolved the structure of the DBD G245S-mp53 in complex with DNA. Thus, to gain more information about this pivotal cell cycle protein, computational molecular modeling tools, such as protein dynamics analysis and/or thermodynamic properties at the atomic spatial resolution and microsecond temporal evolution, can be used. Demir and coworkers in 2011 and Koulgi et al. in 2013 computationally analyzed several hotspot mutants including the G245S-mp53 structural mutant [22,23]. In the former study, the overall protein flexibility of the apo wt-p53 DBD was compared to R175H, G245S, R248Q, R249S, R273H and R282W hotspot mutants through the clustering of molecular dynamics (MD) trajectories. The metric used to evaluate the protein flexibility was the number of clusters obtained by certain RMSD cutoff criteria. Among the several hotspot mutants considered, G245S-mp53 was also simulated. It showed a higher number of clusters with respect to the wt protein suggesting that the p53 hotspot mutations increase the flexibility of the p53 core domain, which indicates thermodynamic instability in agreement with experimental studies [18]. In the latter study by Koulgi and coworkers, the G245S-mp53 bound to DNA was analyzed using quantum and molecular mechanics simulations and was compared to the wt-p53-DNA complex. Furthermore, the free energy of binding (BE) between the p53 DBD and DNA, based on MD simulations, was calculated. The mutant showed a slightly higher BE during a 30 ns trajectory. Hydrogen bonding also decreased and was estimated to occupy only 20% of the simulation time.

In our study, we carried out molecular dynamics simulations on both the wt-p53 and G245S-mp53 mutant in complex with DNA and in their apo form (not bound to DNA) to understand the structural effects of the G245S mutation. We performed comparative conformational analysis between the wt and mutant proteins. Functional Mode Analysis (FMA) was also used to identify the collective atomic motions related to the fluctuations occurring in the mutated region. Clustering was used to identify representative conformations of the p53 dimer in complex with DNA. Furthermore, the binding free energy of the dimer with DNA was evaluated by means of the Molecular Mechanics Generalized Born Surface Area (MMGBSA) method. Our results have shown agreement with experimental data and demonstrated conformational change in the mutant p53 in both cases: DNA bound and apo forms.

## 2. Results

### 2.1. Conformational Analysis

#### 2.1.1. Apo p53 Proteins 

**Figure 1:**
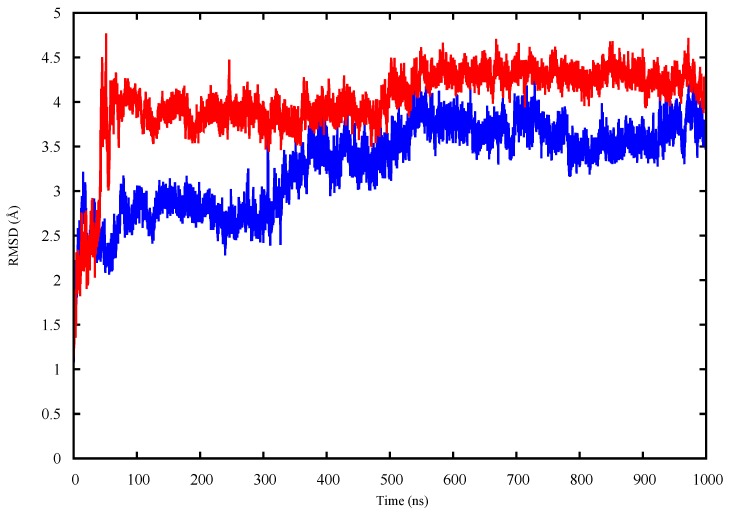
The RMSD plot of the backbone heavy atoms of both wt-p53 (blue) and G245S-mp53 (red) apo monomers over 1 μs of MD simulations.

The apo p53 proteins were simulated using MD for 1 μs. The root mean square deviations (RMSD) of the proteins’ backbones were calculated during the simulation, compared to their starting structures, in order to assess the proteins’ equilibration. The RMSD plots of the apo p53 monomers (Figure 1) show that the RMSD values plateau after 550 ns for both proteins.

**Figure 2:**
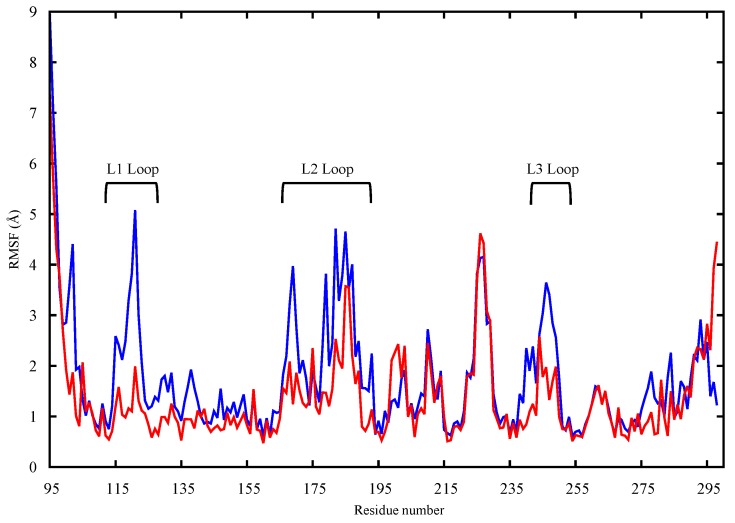
The RMSF plot of the backbone heavy atoms of the apo monomers of wt-p53 (blue) and G245S-mp53 (red). The loops L1, L2 and L3 are indicated.

The protein structures obtained in the last 450 ns of simulation were, therefore, used to assess the dynamic and structural differences between the wt and mutant proteins by means of calculating the root mean square fluctuation (RMSF) of the individual protein residues. For the apo protein structures, the proteins’ backbones were aligned and the RMSF of the backbone atoms for all residues were evaluated and compared. In Figure 2, the wt (blue) and mutant (red) monomers show an overall difference in terms of dynamic behavior as reflected by the different patterns of the RMSF of the different protein residues. The differences in fluctuations are shown in the critical L1 loop of wt-p53 (RMSF of 5 Å) that interacts with the major groove of the DNA and in Lys120 that makes a sequence-specific contact with guanine and interacts with the phosphate backbone of the DNA through its amide (backbone) nitrogen [9]). On the other hand, loop L1 in the G245S mutant has lower RMSF values suggesting that this region becomes less flexible. The loss of the loop L1 flexibility may affect the binding of the mutant to DNA and thus reduce the tumor suppressor activity of the protein. Further, loops L2 and L3 as well as helix H1 are other regions showing higher RMSF values in the wt-p53 compared to the mutant.

The higher fluctuation in the three above-mentioned regions could be explained by the fact that the residues pertaining to the DNA binding regions sample the space to find the DNA molecule, bind to it and finally stabilize it. In loop L3, a key role in DNA binding is played by Arg248 which, in wt-p53, anchors the protein’s DBD to the minor groove of the DNA as shown in the minimized structure of the p53 dimer complexed with DNA (see the Supplementary Data).

**Figure 3:**
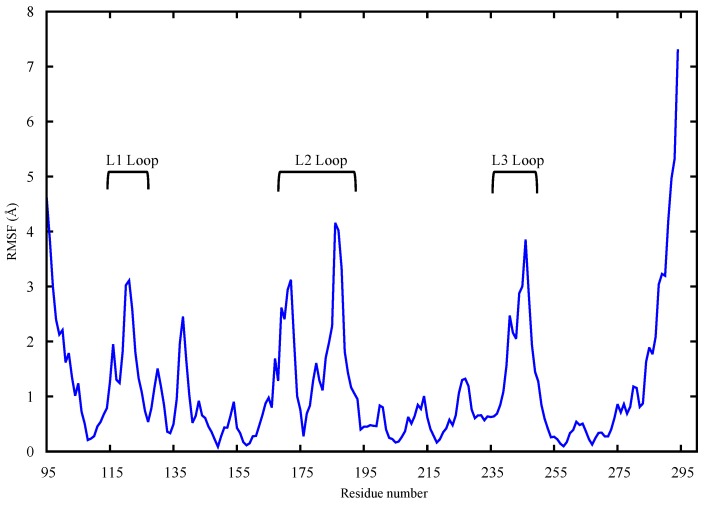
A plot of the RMSF of apo wt-p53 backbone calculated over all the MD trajectory filtered on the ewMCM. The loops L1, L2, L3 are labeled.

Given that a mutation in L3 causes loss of flexibility in L1 and L2, it may be of interest to evaluate existing correlations among the above-mentioned protein areas. Functional Mode Analysis (FMA) was therefore employed, as explained in the method section. In greater detail, FMA allowed the characterization of the contribution of individual PCA vectors to RMSD of loop L3, yielding a single vector, which drives the loop L3 fluctuation mode, referred to as the ensemble-weighted maximally correlated motion (ewMCM). The analysis of the MD trajectory filtered on the ewMCM, enabled the identification of the residues, which are maximally correlated with the fluctuation of L3 (Figure 3). The residue that contributes the most to the whole loop L3 (residues 239–251) fluctuation is G245 due to the inherent flexibility of the glycine amino acid. A high level of correlation was found between loops L2 and L3 as well as loop L3 and helix H1 (residues 163–178). A slightly lower, but significant correlation was also found between loops L3 and L1 (residues 112–124). These findings suggest collective atomic motions among key residues of p53 located at the DNA binding interface. The high RMSF in loops L3, L2 and L1 of wt-p53 (Figure 2) and the FMA outcomes suggest a correlation between these crucial DNA binding loops. Cross-validation of the maximally correlated motion is shown in the Supplementary Data.

#### 2.1.2. p53 Dimers in Complex with the DNA

**Figure 4:**
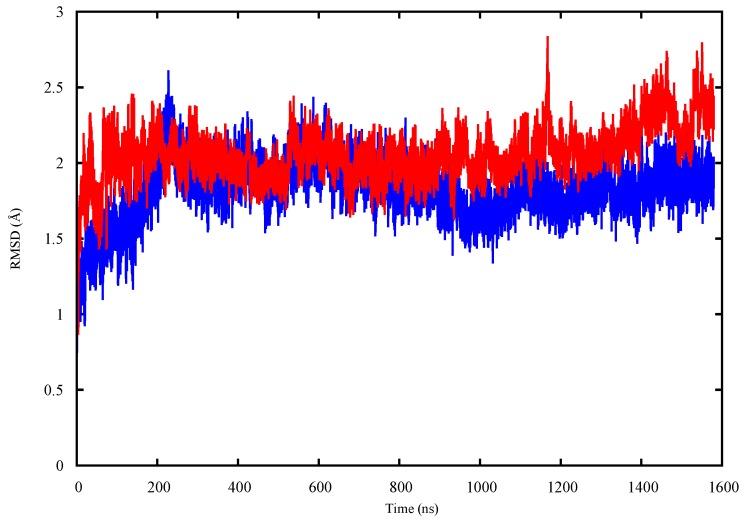
A plot of the RMSD of monomers B of the p53-dimer in complex with DNA; wt-p53 (blue), G245S-mp53 (red).

**Figure 5:**
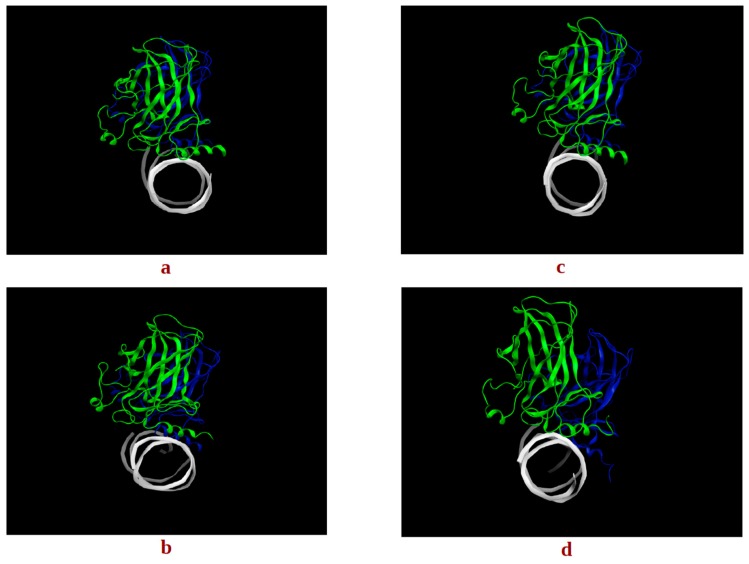
The centroids of the two most populated clusters, (**a**) wt-1 and (**b**) wt-2 of the wt-p53 dimer and (**c**) mt-1 and (**d**) mt-2 of the G245S-mp53 dimer. The rotation of monomer B in the mutant is evident in mt-2 in monomer B (blue) relative to monomer A (green).

Figure 4 shows the RMSDs of the two monomers of the G245S-mp53 dimer, complexed with DNA, compared to the corresponding wt-p53 dimer-DNA complex. The RMSDs of the backbone atoms of all the p53 monomers were calculated compared to their positions at the start of the simulation; the RMSD is reasonably stable after about 500 ns. The analysis of the trajectories of the p53 dimer-DNA complexes demonstrated evidence of rotation of monomer B relative to DNA in the mutant protein models (Figure 5).

**Figure 6:**
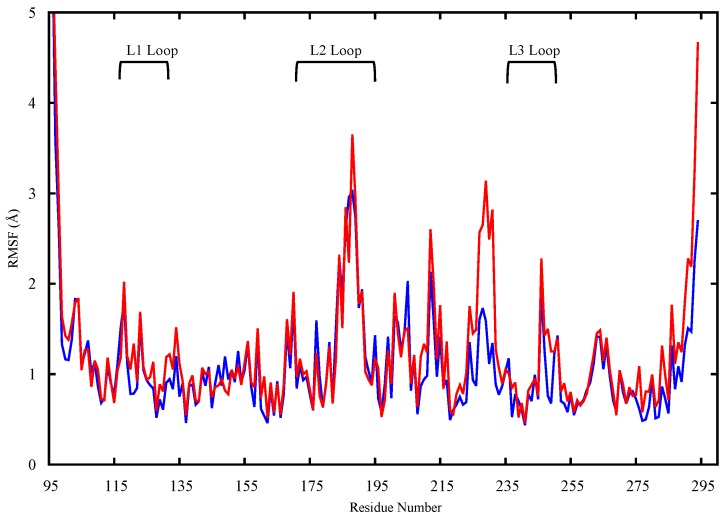
The RMSF plot for the backbone atoms of monomer B in the p53 dimer. Residues Glu224, Val225 and Gly226 have higher fluctuations in G245S-mp53 compared to wt-p53.

**Figure 7:**
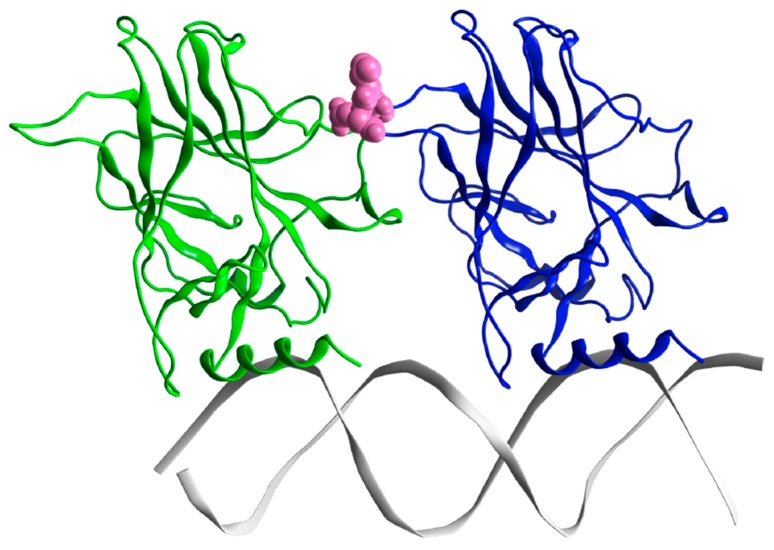
The minimized structure of the wt-p53 dimer in ribbon representation. Monomer A is in green, monomer B in blue and the DNA is grey. The magenta spheres are residues Glu224, Val225 and Gly226, which are at the interface between the two p53 monomers. The loop L1 and helix-2 in monomer B interact with the major groove of DNA, while in monomer A they interact with the DNA terminus.

To highlight the dynamic differences and thus potential structural differences between the wt and G245S-mp53, the RMSFs of the proteins were calculated for the equilibrated part of the trajectory. The RMSF plots of the backbone atoms for monomers B from both the wt/mp53-DNA complexes are shown in Figure 6. Residues E224, V225 and G226 have higher fluctuation in the mutated monomer B compared to the wt. These residues in the wt-p53 dimer are located in a turn that interfaces with the DBD of monomer A as shown in Figure 7.

To assess the overall p53 dimer-DNA conformations sampled during the dynamic simulation, for both the wt and G245S-mp53, an RMSD-based clustering was performed on the equilibrated trajectory frames. The centroid-linkage, average-linkage and complete linkage algorithms were used to perform the RMSD-based structural analysis via clustering. The Davies-Bouldin (DBI), pseudo F-statistic (pSF) and the ratio of the sum of squares regression to the total sum of squares (SSR/SST) clustering metrics were calculated for the last 525 ns of simulation (data not shown). They were also used to assess the clustering quality of the average-linkage algorithm used and to guide the choice of the best cluster [22,24]. As the choice of the optimum number of clusters is usually made to correspond to a local minimum in the DBI value and a plateau in SSR/SST [24], a count of four clusters was chosen. The largest two of the four clusters contained 55% and 36% of all frames, respectively. The last two clusters contained a negligible number of frames as expected for MD simulations that sample the conformational space according to the Boltzmann’s distribution. The same procedure was followed for the protein residues of the G245S-mp53 dimer-DNA complex on the last 1.2 μs (~1200 frames). The optimal number of clusters chosen was 3. Note that 59% and 40% of all frames were found in the two most populated clusters. In the case of the wt proteins, the p53 monomers had similar alignment to the DNA in the two representative structures of the most populated clusters (Figure 5). While the representative structure of the most populated cluster for the mutant proteins had a similar orientation to the DNA as the wt, the second most populated cluster showed misalignment between the two p53 mutant monomers. 

### 2.2. Binding Free Energy (BE) Analysis

For a more quantitative analysis of our results, the total and per-residue BE between the p53 dimers and DNA were evaluated. Indeed, the BE of the two monomers, A and B, to DNA can be evaluated independently. This is reasonable because the monomers can independently bind to the DNA [25].

The BE (ΔΔ G_0_) between p53 and DNA was evaluated using the molecular mechanics generalized Born surface area (MMGBSA) approach. For both the wt and mutant p53-DNA complexes, single equilibrated MD simulations were used to determine the energy values. The total binding energies between the protein dimers and the DNA for both the wt and mutant p53 are shown in Table 1. The calculated BE between the wt-p53 dimers and DNA was −100 kcal/mol (with a standard deviation (SD) of 17 kcal/mol). The G245S-mp53 dimers were calculated to have a lower BE with DNA of −129 kcal/mol (SD of 22 kcal/mol). The binding free energies between the individual monomers to DNA were also evaluated (Table 1). For the wt-p53, monomer A had a BE to DNA of −60 kcal/mol (SD of 15 kcal/mol) while the G245S-mp53 had a comparable BE to DNA of −55 kcal/mol (SD of 13 kcal/mol). For monomer B, however, the BE of the wt-p53 to DNA was −33 kcal/mol (SD of 12 kcal/mol), while for G245S-mp53 the BE was −70 kcal/mol (SD of 20 kcal/mol).

**Figure 8:**
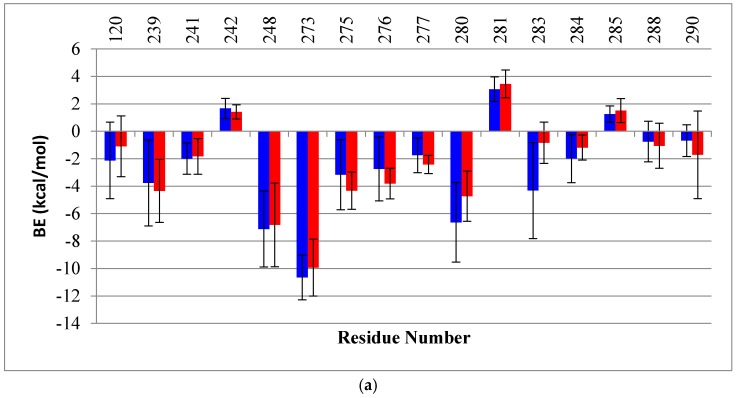

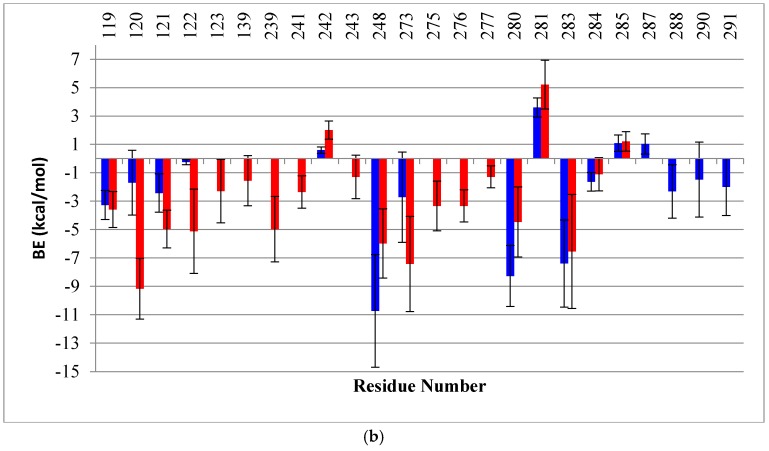
A histogram of the per-residue BE decomposition for (**a**) monomers A and (**b**) monomers B, each in complex with DNA for both the wt-p53 (blue) and G245S-mp53 (red). Only the residues with BE higher or lower than 1 kcal/mol are reported. The *x*-axis represents the residue numbers, the *y*-axis represents the BE and the bars represent SD. A more pronounced redistribution of BE is observed for monomer B.

We also calculated the BE decomposition of the individual contributing residues. When comparing the per-residue decomposition, the residues of the wt monomers A had BE’s very similar to those of the mutant. The plots in Figure 8 show BE again in residues R280 (2 kcal/mol) and Arg283 (4 kcal/mol), which are both involved in the interaction with the DNA major groove. In the case of monomer B, an evident redistribution in the DNA binding residues can be seen clearly for the mutant (red bars) compared to the wt (gray bars). The residues located in the loop L3 (239–250), which are near the mutation, show an overall redistribution of binding energies: R248, which had the lowest BE in the wt to DNA (−12 kcal/mol), showed a BE increase of about 6 kcal/mol in the mutant. Moreover, the mutant had a slight repulsion with residue C242. In addition, new interactions were formed between the DNA and residues C275, A276 and C277 of monomer B of the mutant but not wt-p53. The residues in loop L1 (114–124) of the mutant also had new DNA contacts with residues S121, V122.

In general, for monomer B of G245S-mp53, an increase in interactions is observed in the proximity of the major groove of the DNA, except for the key DNA interacting residue R280. Near the minor groove of DNA, R248, which is also an important DNA interacting residue, lost some of its affinity to DNA. This demonstrates destabilization of the DNA binding in the region near the mutation, but an increase in the affinity of loop L1 and other residues binding near the major groove of the DNA.

The BE’s between the individual protein monomers were also calculated. Our results show that the wt monomers bind with a ΔΔG_0_ of −3.6 kcal/mol (SD of 7 kcal/mol) while the mutant had a corresponding ΔΔG_0_ of only −2 kcal/mol (SD of 7 kcal/mol).

## 3. Discussion

G245S-mp53 is a structural p53 mutant, in other words, a missense mutation produces structural perturbations within the DBD [26], and thus may indirectly affect the pivotal binding of p53 with the consensus site of the DNA, which is essential to maintaining the transcriptional activity of p53. Furthermore, p53 binds to DNA as a tetramer composed of four identical subunits [27], consisting of a dimer of dimers located symmetrically on the consensus site [8,9]. A stabilized G245S variant (quadruple mutant) of the p53 core domain has been resolved by Joerger and his team [28]. It is a multi-site mutant of the p53 and not a single point mutation protein. Moreover, the dynamic behavior of the mutant cannot be observed using X-ray crystallography. In fact, crystal structures are not able to capture the wt protein’s inherently unstable dynamic core domain that is known to have a melting temperature of about 42–44 °C [19]. This is even more true for oncogenic mutations that are thought to inactivate the native protein function by destabilizing or distorting the wt-p53 core domain [29]. Thus, computational simulations such as MD can be useful to complete the experimental results and explore new venues of this complex protein in both dynamic and thermodynamic terms.

The ultimate goal of our research is to generate, by means of in silico molecular modeling, a 3D structure of G245S-mp53 and develop detailed insights into its behavior when it is in the apo form or in complex with DNA. HSQC NMR spectroscopy can be used to experimentally detect the structural effect of a mutation on p53 [30]. In a study to compare the structures of the wt and G245S-mp53, Wong and coworkers used NMR spectroscopy [20]. They only found localized chemical shifts in the G245S-mp53, which indicates that the overall tertiary structure of the protein is similar to the wt-p53. Briefly, they found chemical shifts in the residues located in loops L2 and L3 as well as the terminal residues of the β-strands 4, 9 and 10. These differences are also reflected by the dynamic behavior shown in our study by the RMSF of the apo monomers backbone atoms (Figure 2); higher fluctuations in wt-p53 loops L1, L2, and L3 are evident.

FMA results identified a collective motion of loops L1, L2, helix H1 of wt-p53 that are maximally correlated to the fluctuation of loop L3, at which the G245S lies. FMA can identify motions of the protein that are not evident in other well-established methods such as Principal Component Analysis (PCA) and Normal Mode Analysis (NMA) [31,32]. PCA and NMA extract the collective motions with the largest contribution to the variance of the atomic fluctuation and the lowest frequency modes, respectively, whereas FMA accounts for collective modes distributed over a number of normal or PCA modes. As different motions of the protein could be related to the functional quantity (RMSD of loop L3 in our case), the generated frames of the protein are used to estimate the most probable collective motion that is responsible for the determined variation in the functional quantity. This is called ensemble-weighted maximal correlated motion (ewMCM). In the RMSF plot calculated over the MD trajectory filtered on the ewMCM (Figure 5), the high peak detected in G245 may be explained by the inherent ability of glycine to adopt unusual backbone dihedral angles that allows for a higher flexibility in the wt protein. However, when the residue is mutated to serine, there is a restriction on the allowed dihedral angles and thus the neighboring residues undergo dynamic stiffening (as observed in the RMSF plot of Figure 4). Moreover, residue C242 in the L3-loop, coordinates a Zn^2+^ ion along with residues C176 and C179 in the L2-loop. Our results suggest that after the missense mutation of glycine to serine and its resultant dynamic stiffening on loop L3, a “domino effect” follows which causes a decrease in the L2-loop dynamics when compared to the wt-p53. Figure 4 also shows that the L1-loop of G245S-mp53 has lower fluctuations when compared with the wt protein. While it was difficult to find evidence of correlation between the L1 and L3 loops by visualizing the MD trajectory of the proteins, FMA data highlight a collective motion involving loops L1 and L3.

The dynamics of the central β-strands scaffold (res. 110–112; 141–146; 156–163; 195–198; 204–207; 214–219; 230–236; 251–258; 264–273) in the mutated apo protein are similar to that of the wt apo protein. However, reduced flexibility is observed in loops L1, L2 and L3 where the key residues K120 (L1), S241 (S8–L3) A248 (L3) are located and are known to interact directly with the DNA molecule [9]. This reduction of fluctuations in G245S-mp53 key regions may be the reason for defective binding with DNA and thus p53 inactivation. More specifically, R248 in wt-p53, which is close to the G245 mutation site and is the residue with the most gain in BE with DNA upon mutation, is able to protrude into the minor groove of the DNA molecule resulting in favorable electrostatic interactions between the positively charged guanidinium group of R248 and the negatively charged DNA backbone. Furthermore, the minor groove adjacent to R248 is compressed and its bases are buckled so that the side chain of R248 makes three water mediated hydrogen bond contacts with the DNA molecule.

The evident differences in the dynamics of the key regions in p53, which are known to interact with DNA, have led us to also simulate the wt and G245S-mp53 proteins each in complex with DNA. To the best of our knowledge, there are no experimentally resolved structures of the G245S-mp53-DNA complex. To create this model, we used the crystal structure of wt-p53-DNA complex (PDB ID: 4HJE [8]), and virtually mutated residues G245 to serine in each monomer. We simulated the p53 dimer, both the wt and mutant proteins, in complex with DNA for more than 1.5 μs using MD to reach a reasonable equilibrated complex structure.

For analysis, we assume monomer B is a better model for the p53-DNA interaction since loop L1 and helix-2 of monomer A interact with the terminus of the DNA rather than its major groove. The RMSF plots of monomer B in the G245S and wt-p53 DNA complexes are shown in Figure 6. It is evident that the high residue fluctuations observed in the monomers in loops L1, L2 and L3 are not observed in the DNA complexed proteins. This is expected since the G245S-mp53 3D-model was created by a virtual single point mutation on the wt-p53-DNA complex. The wt protein, when in complex with the DNA, is expected to be more stable and hence the high fluctuations that were evident in the apo-monomers would decrease in the p53-DNA [33,34]. Furthermore, the overall structure may assume a different conformation, even far from the DNA binding site, after DNA binding as confirmed by recent studies [35]. Nevertheless, our model shows that G245S-mp53 has higher fluctuations in the residues between β-strands 7 and 8 (residues 224, 225 and 226), which are normally located at the interface of the two protein monomers in the wt-p53. This relatively higher fluctuation in the mutant protein indicates a possible interruption in the monomer interaction and the exposure of these residues to the solvent. To further investigate this issue, the trajectory analysis showed a relative displacement between monomers A and B. This first qualitative result suggests that the mutant dimers undergo rearrangement upon DNA binding resulting in a dimer distortion without structural unfolding in agreement with experimental studies, which state that there is very little unfolding in G245S-mp53 that is not as structurally destabilized as other mutants [36]. To confirm the observation of monomer displacement, RMSD based clustering was performed to extract representative structures of all the conformational space sampled during the MD simulation. A comparison between the centroids of the most populated clusters has confirmed our observation. Indeed, Figure 5 shows symmetrical arrangement of the two protein monomers on the DNA in the wt-p53 for almost the whole trajectory. However, there is distortion in the alignment of G245S-mp53 monomers in its second representative structure, which presents about 50% of the trajectory.

Interestingly, a previous study has shown evidence that mutations in the p53 tetramerization domain can inactivate the wt protein in a manner similar to that seen in the mutated DBD [37]. It is possible that the distortion in the dimer structure observed in G245S-mp53 might not allow the proper protein tetramerization and hence decreases or disables the tumor suppressor activity of the p53 [38].

To further understand the relationship between the G245S mutation and its evident consequence of monomer reorientation, a quantitative evaluation of the binding free energies between the individual monomers as well as between the dimers to the DNA was obtained by means of MMGBSA. This method combines the molecular mechanical energies with the continuum solvent approach where the electrostatic contribution to the solvent free energy is evaluated by the generalized Born method. Koulgi and his team previously employed a similar evaluation on a 30 ns trajectory [23]; the evaluated BE of G245S-mp53 to DNA ranged from −65 to −40 kcal/mol. In our work, longer equilibrated trajectories (the last 1 μs) were used to identify the residues with lost or new interactions with DNA. However, we assume that the results for monomer B are more reliable as it is better centered on the DNA as explained earlier (Figure 7). The plots of the per-residue BE decomposition of monomer B to DNA showed complete reorganization of the residues interacting with DNA. As shown in Figure 8, residues close to the G245S mutation such as R248 and C242 have a BE increase of about 6 kcal/mol and 2.5 kcal/mol, respectively, when compared to wt-p53. These results suggest a conformational rearrangement in the region close to G245S mutation. Consequently, the conformational rearrangement near the G245S mutation results in disorientation of the p53 monomers bound to DNA with a consequent increase in the BE of the key DNA binding residue R280 of about 4.5 kcal/mol and gain of BE of other residues such as R280 and R283 to the DNA along with the formation of non-canonical DNA interactions such as residues 122, 123 and 139.

The overall effect of BE redistribution results in misalignment between the monomers that probably leads to destabilization of the dimer formation and hence an expected destabilization in the tetramer, which would lead to the decrease or loss of the transcriptional activity of p53. The binding energies between the protein monomers on the same site of the DNA are high. This is expected because the tetramerization domain has not been simulated, however a stronger interaction may be found between opposite DBD monomers bound on the same half site DNA [39].

Finally, from the outcome of our work emerges the suggestion that the L3-loop stiffening, which also affects the dynamics of L1 and L2, may be responsible for the reduction of p53 affinity to DNA in the mutated protein. Despite the fact that we simulated the protein for about 1.5 μs, classical MD may still not be able to capture the whole protein dynamics due to the method’s limited sampling capability. Nonetheless, our MD results clearly highlight the G245S mutation as responsible for a dimer reorganization and distortion not observed in the wt-p53. Starting from our findings, enhanced sampling techniques, able to better sample the phase space in simulations of protein-protein and protein-nucleic acid molecular systems, together with dimensionality reduction [40,41,42,43,44,45] might provide further insights into the p53-DNA complex dynamics [46].

## 4. Materials and Methods 

### 4.1. 3D Structure Preparation

For the DNA bound proteins, the initial atomic coordinates of the wt-p53 DBD-DNA tetramer complex were obtained from the x-ray crystallographic structure with PDB ID: 4HJE [8]. In this structure, the DBD binds to DNA response elements composed of two decameric half-sites separated by 1bp (5′-TCACAAGTTAGAGDCAAGCCT-3′) [47]. The crystal waters that mediated interactions between p53 and the DNA were retained. Only two of the four p53 monomers, which bind to DNA on the same side, as well as the bound DNA were included so our simulations could be carried out within a reasonable time. The starting structure of the G245S-mp53 dimer in complex with DNA was obtained from the wt p53 dimer-DNA complex by virtually mutating glycine 245 to serine using PyMol [48]. The protein structures were corrected using Molecular Operating Environment (MOE) software [49]. The protonation states of both the mutant and the wt were calculated using 3Dprotonate [50] in MOE at pH 7, with 0.1 M sodium chloride concentration at 310 K. Moreover, the Zn^2+^ coordinating residues (Cys176, His179, Cys238, Cys242) were deprotonated. Each system, comprised of a p53 dimer in complex with DNA and its co-crystallized water molecules, was solvated in a TIP3P octahedral water box. The boundaries of the box were chosen to provide a water buffer of 12 Å around the complex along each dimension (a total of 26,178 explicit water molecules). Furthermore, chloride and sodium ions were added to simulate a physiological ionic concentration of 0.1 M. The ions were positioned to replace water molecules having the highest electrostatic energies on their oxygen atoms. For the apo p53 monomers, the initial atomic coordinates for the apo wt-p53 monomer were obtained from the NMR-resolved structure with PDB ID: 2FEJ [18]. The wt and G245S-mutant apo p53 monomers were both prepared in the same manner described for the dimers above. All the systems were solvated and prepared using AmberTools14 [51].

### 4.2. Molecular Dynamics Simulation

Similar to our previous study [52], the water molecules were minimized for 3000 steps of steepest descent followed by 2000 steps of conjugate gradient minimization; heavy restraints of 100 kcal/mol·Å^2^ were placed on the proteins and DNA [51]. The restraints were then removed and a minimization of the whole system was performed through a series of 2500 steepest descent followed by 2500 conjugate gradient steps. Further, the system was gradually heated up to 310 K in 200 ps and maintained at 310 K for another 100 ps under constant volume conditions (NVT). The particle-mesh Ewald procedure was used to handle long-range electrostatic interactions with a 10 Å cut-off. The Langevin thermostat was used with a time collision frequency of 2 ps [53]. Hydrogen atoms were constrained by the SHAKE algorithm and the heavy atoms of the proteins backbone and DNA were heated using 2 kcal/mol·Å^2^ restraints [54]. These restrains were then gradually reduced and the p53 dimers-DNA complexes were simulated for 1.5 μs while the p53 monomers were simulated for 1 μs. Before running the production in the MD simulation, the systems were assessed by means of their potential, kinetic and total energies, the temperature, pressure and the density of the systems over the simulation times (data not shown). All simulations were performed using the Amberff14SB force-field [55].

### 4.3. Conformational Change Analysis

#### 4.3.1. RMSD and RMSF 

The system’s equilibrium and the dynamic fluctuation of p53 residues were analyzed by means of calculating mass-weighed root mean square deviation (RMSD) of the backbone atoms and the residues root mean square fluctuation (RMSF), respectively. These two metrics are measures of distance variation of the protein atoms during the simulation and were evaluated using AmberTools14 [51].

#### 4.3.2. FMA

An ensemble of structures obtained from the MD simulations was used to underline the collective motions directly related to loop L3 fluctuation (functional quantity). We used a set of protein structures to find the collective protein motion that is maximally related to the “functional quantity” e.g., the RMSD of the L3 loop. In other words, we performed FMA on the whole simulated MD trajectory of the apo p53 monomers to highlight the collective motions directly correlated to the fluctuation of the region involved in DBD mutation as done in previous literature studies [43,44,56] More specifically, considering the variable of interest as a linear function of principal components, the maximally correlated vector was obtained by maximizing the Pearson coefficient to quantify the contributions of the individual PCA vectors to the fluctuation of the variable of interest [56]. 

#### 4.3.3. Clustering 

RMSD-based clustering was used to extract protein structures to represent the overall protein flexibility and thus provide means of examining the sampled conformations during the MD simulation. RMSD based clustering was obtained with bottom up algorithms such as average-linkage, single linkage and centroid-linkage methods, all implemented in the utility of AMBER14. We used the equilibrated portions of the MD trajectories for RMSD based clustering using the average-linkage algorithm in AmberTools14 [51]). Several studies have discussed and validated the use of hierarchical algorithms in MD simulations [22,24]. Before clustering, a mass-weighted RMSD fit of the heavy atoms of the backbone to the protein starting structure was performed. To assess the clustering quality and find the optimum number of clusters, three clustering metrics: the Davies-Bouldin index (DBI) [57], the pseudo F-statistic [58], and the sum of square regression-sum of total sum of square ratio (SSR/SST), were plotted for each cluster count. The optimum number of clusters occurs at a local DBI value minimum, a local pSF value maximum and when the SSR/SST ratios plateau [24]. The centroid structures (the structures having the smallest RMSD relative to all the other members of the same cluster) of each cluster were extracted and used for comparative analyses.

### 4.4. Binding Energy Calculation

The binding free energies of p53 to DNA and the p53 dimers to each other, for both the wt and the G245S-mp53 mutant were calculated using the Molecular Mechanics Generalized Born Surface Area (MMGBSA) [59,60]. The free energy was thus calculated with the software AMBER14 [51]. The BE’s between p53 (receptor) and DNA (ligand) were calculated for the p53-DNA complexes for the equilibrated structures as:

ΔG_bind_ = ΔH − TΔS ≈ ΔE_MM_ + ΔG_sol_ − TΔS, (1)

where ΔE_MM_ is the molecular mechanic energy in the gas phase and includes the bond, angle and dihedral energies as well as electrostatic and van der Waals energies:

ΔE_MM_ = ΔE_inter_ + ΔE_elec_ + ΔE_vDW_, (2)

and ΔG_sol_ is the solvation free energy given by the sum of the electrostatic solvation energy or polar Energy and a non-electrostatic solvation component (non polar contribution). Here, the electrostatic solvation contribution is evaluated using the Generalized Born (GB) model, while the non-polar contribution is approximated by the solvent accessible area (SASA):

ΔG_sol_ = ΔG_GB_ + ΔG_SA_, (3)

The conformational entropy change −TΔS can be computed by normal-mode analysis. However, since we were interested in the relative rank of binding energies rather than the absolute values and since the calculation of the entropy term is computationally demanding, the entropy term was assumed to be constant. Therefore, we used the single trajectory approach to evaluate the BE between the proteins and DNA and between the individual p53 monomers in the p53 dimer complexes by means of the thermodynamic cycle:

ΔG°_bind,solv_ = ΔG°_p53_ − _DNA,vacuum_ + ΔG°_p53_ − _DNA,solv_ − ΔG°_solv,DNA_ − ΔG°_solv,p53_ (4)

## Figures and Tables

**Table 1:** A table of the BE between the p53 dimer and the DNA, the BE between the p53 monomer A and B, the BE between monomer A and the DNA and the BE between monomer B and the DNA.

**BE of the p53 Dimer to DNA**			**BE of p53 Monomer A to B**		
	**BE (kcal/mol)**	**SD (kcal/mol)**		**BE (kcal/mol)**	**SD (kcal/mol)**
wt-p53	−100	17	wt-p53	−4	7
G245S-mp53	−129	22	G245S-mp53	−2	7
**BE of p53 Monomer A to DNA**			**BE of p53 Monomer B to DNA**		
	**BE (kcal/mol)**	**SD (kcal/mol)**		**BE (kcal/mol)**	**SD (kcal/mol)**
wt-p53	−60	15	wt-p53	−33	12
G245S-mp53	−55	13	G245S-mp53	−70	20

## Supplementary Materials

Supplementary materials are available online.

## References

[1] Vogelstein B., Lane D., Levine A.J. (2000). Surfing the p53 network. Nature.

[2] Goldstein I., Marcel V., Olivier M., Oren M., Rotter V., Hainaut P. (2011). Understanding wild-type and mutant p53 activities in human cancer: New landmarks on the way to targeted therapies. Cancer Gene Ther..

[3] Wang Z., Sun Y. (2010). Targeting p53 for Novel Anticancer Therapy. Transl. Oncol..

[4] Bykov V.J.N., Wiman K.G. (2014). Mutant p53 reactivation by small molecules makes its way to the clinic. FEBS Lett..

[5] Kogan S., Carpizo D. (2016). Pharmacological targeting of mutant p53. Transl. Cancer Res..

[6] Olivier M., Eeles R., Hollstein M., Khan M.A., Harris C.C., Hainaut P. (2002). The IARC TP53 database: New online mutation analysis and recommendations to users. Hum. Mutat..

[7] McLure K.G., Lee P.W.K. (1998). How p53 binds DNA as a tetramer. EMBO J..

[8] Chen Y., Zhang X., Dantas Machado A.C., Ding Y., Chen Z., Qin P.Z., Rohs R., Chen L. (2013). Structure of p53 binding to the BAX response element reveals DNA unwinding and compression to accommodate base-pair insertion. Nucleic Acids Res..

[9] Cho Y., Gorina S., Jeffrey P.D., Pavletich N.P. (1994). Crystal structure of a p53 tumor suppressor-DNA complex: Understanding tumorigenic mutations. Science.

[10] Chen Y., Dey R., Chen L. (2010). Crystal Structure of the p53 Core Domain Bound to a Full Consensus Site as a Self-Assembled Tetramer. Structure.

[11] Duan J., Nilsson L. (2006). Effect of Zn^2+^ on DNA recognition and stability of the p53 DNA-binding domain. Biochemistry.

[12] Hanel W., Marchenko N., Xu S., Yu S.X., Weng W., Moll U. (2013). Two hot spot mutant p53 mouse models display differential gain of function in tumorigenesis. Cell Death Differ..

[13] Xu J., Qian J., Hu Y., Wang J., Zhou X., Chen H., Fang J.Y. (2014). Heterogeneity of Li-Fraumeni syndrome links to unequal gain-of-function effects of p53 mutations. Sci. Rep..

[14] Samowitz W.S., Curtin K., Ma K., Edwards S., Schaffer D., Leppert M.F., Slattery M.L. (2002). Prognostic significance of p53 mutations in colon cancer at the population level. Int. J. Cancer.

[15] Lehmann S., Bykov V.J.N., Ali D., Andreń O., Cherif H., Tidefelt U., Uggla B., Yachnin J., Juliusson G., Moshfegh A. (2012). Targeting p53 in vivo: A first-in-human study with p53-targeting compound APR-246 in refractory hematologic malignancies and prostate cancer. J. Clin. Oncol..

[16] Wassman C.D., Baronio R., Demir Ö., Wallentine B.D., Chen C.K., Hall L.V., Salehi F., Lin D.W., Chung B.P., Hatfield G.W. (2013). Computational identification of a transiently open L1/S3 pocket for reactivation of mutant p53. Nat. Commun..

[17] Zhao K., Chai X., Johnston K., Clements A., Marmorstein R. (2001). Crystal Structure of the Mouse p53 Core DNA-binding Domain at 2.7 Å Resolution. J. Biol. Chem..

[18] Cañadillas J.M.P., Tidow H., Freund S.M.V., Rutherford T.J., Ang H.C., Fersht A.R. (2006). Solution structure of p53 core domain: Structural basis for its instability. Proc. Natl. Acad. Sci. USA.

[19] Hainaut P., Wiman K.G. (2005). 25 Years of p53 Research.

[20] Wong K.B., DeDecker B.S., Freund S.M., Proctor M.R., Bycroft M., Fersht A.R. (1999). Hot-spot mutants of p53 core domain evince characteristic local structural changes. Proc. Natl. Acad. Sci. USA.

[21] Joerger A.C., Ang H.C., Fersht A.R. (2006). Structural basis for understanding oncogenic p53 mutations and designing rescue drugs. Proc. Natl. Acad. Sci. USA.

[22] Demir Ö., Baronio R., Salehi F., Wassman C.D., Hall L., Hatfield G.W., Chamberlin R., Kaiser P., Lathrop R.H., Amaro R.E. (2011). Ensemble-based computational approach discriminates functional activity of p53 cancer and rescue mutants. PLoS Comput. Biol..

[23] Koulgi S., Achalere A., Sharma N., Sonavane U., Joshi R. (2013). QM-MM simulations on p53-DNA complex: A study of hot spot and rescue mutants. J. Mol. Model..

[24] Shao J., Tanner S.W., Thompson N., Cheatham T.E. (2007). Clustering molecular dynamics trajectories: 1. Characterizing the performance of different clustering algorithms. J. Chem. Theory Comput..

[25] Wang Y.U.N., Schwedes J.F., Parks D., Mann K., Tegtmeyer P. (1995). Interaction of p53 with its consensus DNA-binding site. Mol. Cell. Biol..

[26] Soussi T., Lozano G. (2005). p53 mutation heterogeneity in cancer. Biochem. Biophys. Res. Commun..

[27] Chan W.M., Siu W.Y., Lau A., Poon R.Y.C. (2004). How many mutant p53 molecules are needed to inactivate a tetramer?. Mol. Cell. Biol..

[28] Joerger A., Fersht A. (2007). Structure–function–rescue: The diverse nature of common p53 cancer mutants. Oncogene.

[29] Bullock A.N., Fersht A.R. (2001). Rescuing the function of mutant p53. Nat. Rev. Cancer.

[30] Friedler A., DeDecker B.S., Freund S.M.V., Blair C., Rüdiger S., Fersht A.R. (2004). Structural Distortion of p53 by the Mutation R249S and its Rescue by a Designed Peptide: Implications for “mutant Conformation”. J. Mol. Biol..

[31] David C.C., Jacobs D.J. (2014). Principal component analysis: A method for determining the essential dynamics of proteins. Methods Mol. Biol..

[32] Bahar I., Lezon T.R., Yang L.W., Eyal E. (2010). Global dynamics of proteins: Bridging between structure and function. Annu. Rev. Biophys..

[33] Lukman S., Lane D.P., Verma C.S. (2013). Mapping the structural and dynamical features of multiple p53 DNA binding domains: Insights into loop 1 intrinsic dynamics. PLoS ONE.

[34] Ishimaru D., Ano Bom A.P.D., Lima L.M.T.R., Quesado P.A., Oyama M.F.C., De Moura Gallo C.V., Cordeiro Y., Silva J.L. (2009). Cognate DNA stabilizes the tumor suppressor p53 and prevents misfolding and aggregation. Biochemistry.

[35] Lambrughi M., De Gioia L., Gervasio F.L., Lindorff-Larsen K., Nussinov R., Urani C., Bruschi M., Papaleo E. (2016). DNA-binding protects p53 from interactions with cofactors involved in transcription-independent functions. Nucleic Acids Res..

[36] Selivanova G., Wiman K.G. (2007). Reactivation of mutant p53: Molecular mechanisms and therapeutic potential. Oncogene.

[37] Chène P., Che P. (2001). The role of tetramerization in p53 function. Oncogene.

[38] Kamada R., Nomura T., Anderson C.W., Sakaguchi K. (2011). Cancer-associated p53 tetramerization domain mutants: Quantitative analysis reveals a low threshold for tumor suppressor inactivation. J. Biol. Chem..

[39] Ho W.C., Fitzgerald M.X., Marmorstein R. (2006). Structure of the p53 core domain dimer bound to DNA. J. Biol. Chem..

[40] Paciello G., Acquaviva A., Ficarra E., Deriu M.A., Macii E.A. (2011). molecular dynamics study of a miRNA:mRNA interaction. J. Mol. Model..

[41] Grasso G., Tuszynski J.A., Morbiducci U., Licandro G., Danani A., Deriu M.A. (2017). Thermodynamic and kinetic stability of the Josephin Domain closed arrangement: Evidences from replica exchange molecular dynamics. Biol. Direct.

[42] Deriu M.A., Grasso G., Tuszynski J.A., Gallo D., Morbiducci U., Danani A. (2016). Josephin Domain Structural Conformations Explored by Metadynamics in Essential Coordinates. PLoS Comput. Biol..

[43] Deriu M.A., Grasso G., Tuszynski J.A., Massai D., Gallo D., Morbiducci U., Danani A. (2016). Characterization of the AXH domain of Ataxin-1 using enhanced sampling and functional mode analysis. Proteins Struct. Funct. Bioinform..

[44] Grasso G., Deriu M.A., Tuszynski J.A., Gallo D., Morbiducci U., Danani A. (2016). Conformational fluctuations of the AXH monomer of Ataxin-1. Proteins Struct. Funct. Bioinform..

[45] Grasso G., Deriu M.A., Prat M., Rimondini L., Vernè E., Follenzi A., Danani A. (2015). Cell Penetrating Peptide Adsorption on Magnetite and Silica Surfaces: A Computational Investigation. J. Phys. Chem. B.

[46] Bernardi R.C., Melo M.C.R., Schulten K. (2015). Enhanced sampling techniques in molecular dynamics simulations of biological systems. Biochim. Biophys. Acta.

[47] Wei C.L., Wu Q., Vega V.B., Chiu K.P., Ng P., Zhang T., Shahab A., Yong H.C., Fu Y., Weng Z. (2006). A global map of p53 transcription-factor binding sites in the human genome. Cell.

[48] (2017). The PyMOL Molecular Graphics System.

[49] Chemical Computing Group Inc. (2004). Molecular Operating Environment (MOE). Sci. Comput. Instrum..

[50] Labute P. (2008). The generalized born/volume integral implicit solvent model: Estimation of the free energy of hydration using London dispersion instead of atomic surface area. J. Comput. Chem..

[51] Case D.A., Babin V., Berryman J.T., Betz R.M., Cai Q., Cerutti D.S., Cheatham T.E., Darden T.A., Duke R.E., Gohlke H. (2014). Amber14 Reference Manual.

[52] Omar S.I., Tuszynski J. (2015). Ranking the Binding Energies of p53 Mutant Activators and Their ADMET Properties. Chem. Biol. Drug Des..

[53] Loncharich R.J., Brooks B.R., Pastor R.W. (1992). Langevin dynamics of peptides: The frictional dependence of isomerization rates of *N*-acetylalanyl-*N*′-methylamide. Biopolymers.

[54] Ryckaert J.P., Ciccotti G., Berendsen H.J.C. (1977). Numerical integration of the cartesian equations of motion of a system with constraints: Molecular dynamics of n-alkanes. J. Comput. Phys..

[55] Maier J.A., Martinez C., Kasavajhala K., Wickstrom L., Hauser K.E., Simmerling C. (2015). ff14SB: Improving the Accuracy of Protein Side Chain and Backbone Parameters from ff99SB. J. Chem. Theory Comput..

[56] Hub J.S., De Groot B.L. (2009). Detection of functional modes in protein dynamics. PLoS Comput. Biol..

[57] Davies D.L., Bouldin D.W. (1979). A cluster separation measure. IEEE Trans. Pattern Anal. Mach. Intell..

[58] Caliński T., Harabasz J. (2007). A dendrite method for cluster analysis. Commun. Stat. Methods.

[59] Onufriev A., Bashford D., Case D.A. (2000). Modification of the Generalized Born model suitable for macromolecules. J. Phys. Chem. B.

[60] Srinivasan J., Cheatham T.E., Cieplak P., Kollman P.A., Case D.A. (1998). Continuum solvent studies of the stability of DNA, RNA, and phosphoramidate-DNA helices. J. Am. Chem. Soc..

