# Supplementary material for: Insights into the Effect of the G245S Single Point Mutation on the Structure of p53 and the Binding of the Protein to DNA
Source: Molecules. 2017 Aug 16;22(8):1358. doi: 10.3390/molecules22081358 (PMC6152093; doi:10.3390/molecules22081358)
Supplement: Supplementary file 1 [file molecules-22-01358-s001.pdf]

Supplementary Figures

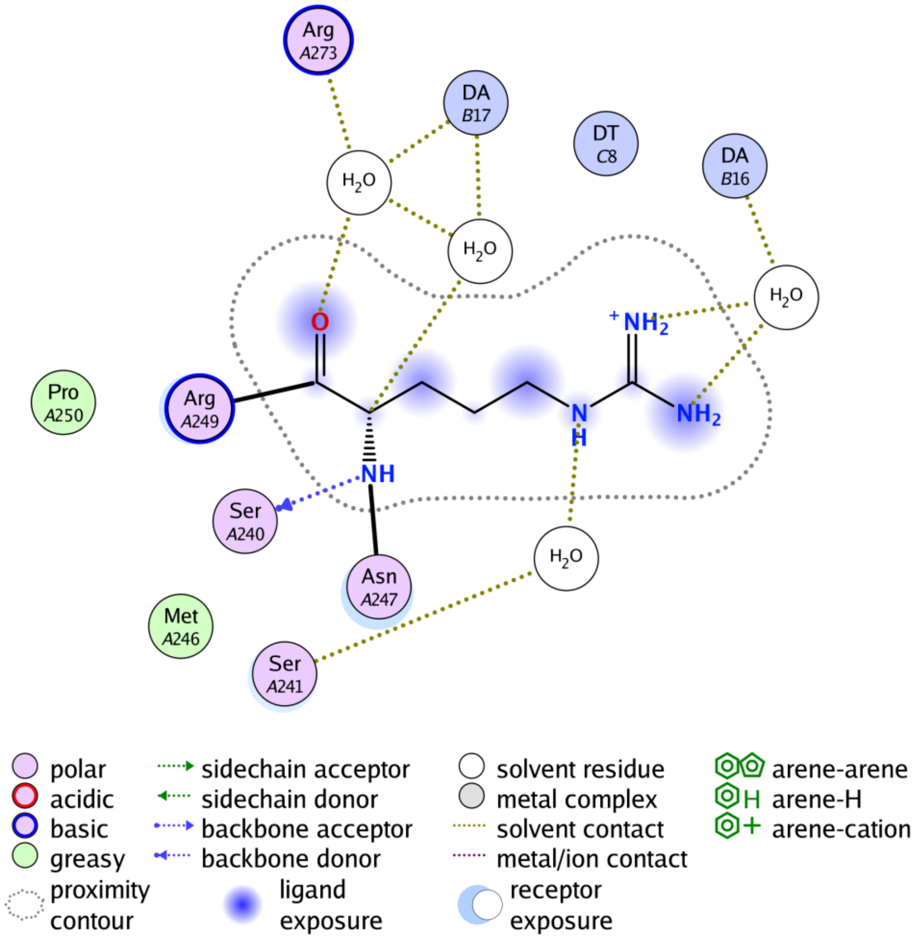

Figure S1: Arg248 in monomer B of wt-p53 makes direct or water mediated contacts with DNA backbone thus retaining its water crystal structures is important.

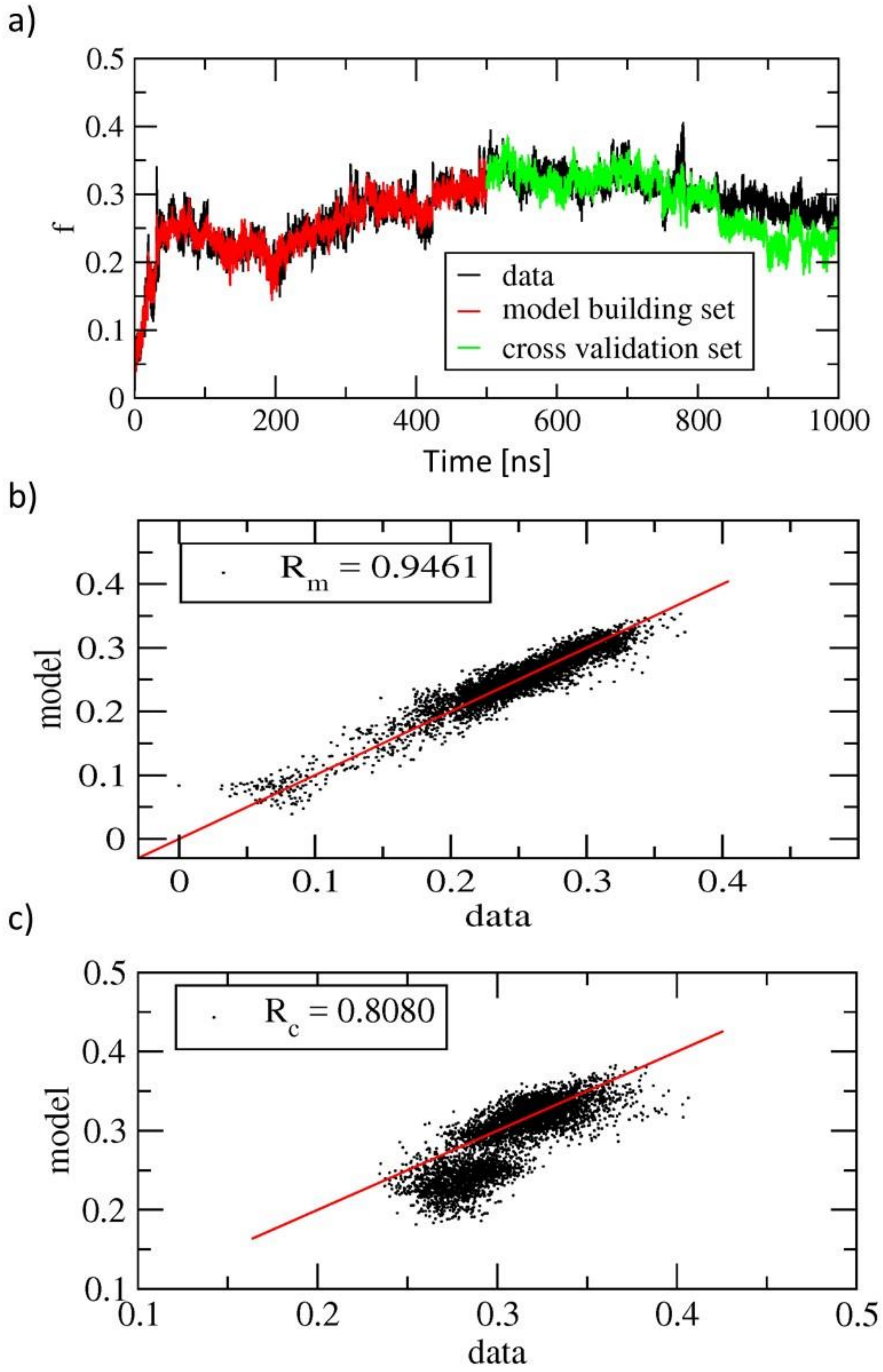

Figure S2: Cross-validation of the maximally correlated motion by FMA employing as functional quantity the RMSD of loop L3 throughout the whole MD simulation. a) Loop L3 RMSD along the MD simulation (black); model in the model building set (red); model in the validation set (green). b) scatter plot (data-model) of the model building with its Rm value , that is the correlation between data and model in the model building set. c) Scatter plot (data-model) of the cross-validation set along with the correlation Rc.
